# Chronic lymphocytic leukemia associated with genetic susceptibility and occupational glyphosate exposure: a case report

**DOI:** 10.3389/fonc.2026.1863949

**Published:** 2026-09-17

**Authors:** Rachel M. Fiegener, Lindsay C. Scally, Raha Abbasian, Abigail L. Warren, James M. Small

**Affiliations:** 1 Rocky Vista University College of Osteopathic Medicine, Parker, CO, United States; 2 Rocky Vista University College of Osteopathic Medicine, Department of Biomedical Sciences, Parker, CO, United States

**Keywords:** chronic lymphocytic leukemia, environmental carcinogenesis, familial leukemia, gene–environment interaction, glyphosate exposure

## Abstract

Chronic lymphocytic leukemia (CLL) is the most common adult leukemia in Western countries, typically diagnosed in older adults, with a median age of 72. Despite its prevalence, the etiology is unclear, and cases at younger ages have been increasingly reported. We present a case of a male diagnosed in his mid-sixties with CLL to underscore the need for further investigation into the poorly understood etiology and management of CLL. The patient presented to his primary care provider for an annual examination, where an elevated white blood cell (WBC) count was first detected. Over the following two years, persistently elevated WBC counts were noted. Based on a peripheral blood smear and flow cytometry, oncology confirmed the diagnosis of CLL. At present, the patient is categorized at Rai Stage 1. Primary management is active surveillance, reflecting the current therapeutic uncertainty in early-stage disease. In this case, the history revealed prolonged occupational exposure to glyphosate-based (Roundup^®^). The patient also had a family history of CLL in his father, who had been exposed to Agent Orange. Although no causal relationship can be inferred from a single case, the co-occurrence of a family history of CLL and occupational glyphosate exposure raises the possibility that multiple risk factors may be relevant to disease development. This case highlights the need for further research into the genetic and environmental factors associated with CLL pathogenesis.

## 1 Introduction

Chronic Lymphocytic Leukemia (CLL) is a lymphoproliferative disorder characterized by the accumulation of atypical B-lymphocytes in the peripheral blood, bone marrow, lymph nodes, and spleen. Patients are initially asymptomatic, but later present with lymphadenopathy, cytopenias, and systemic symptoms consisting of night sweats, unintentional weight loss, fatigue, and early satiety (1). The incidence of CLL increases by age, with a median diagnostic age of 75 and a male-to-female ratio of 1.5–2:1 (2). Diagnosis of CLL depends on the presence of at least 5× 10³/µL aberrant monoclonal B lymphocytes in the peripheral blood with confirmation of CD5, CD19, and CD23 markers via flow cytometry (3).

The heterogeneous nature of CLL makes treatment difficult; some patients experience an indolent course requiring no treatment, while others develop aggressive disease and frequent relapse. The prognosis and treatment plan are dependent on Rai staging, patient fitness, disease status, and B-cell genetic profiling (2). Despite being the most common adult leukemia in the Western world, the etiology of CLL remains poorly understood, particularly in younger populations. Recent studies have investigated the effects of latency, genetic, and environmental risk factors on the development of early B-cell malignancy. Prior research has examined possible associations between environmental exposures, including glyphosate-based herbicides and Agent Orange, with the risk of lymphoid malignancies (4). Other studies have demonstrated an increased risk of CLL among individuals with first-degree relatives affected by hematologic malignancies (5). In this case, we describe CLL in a man in his mid-sixties with both a family history of CLL and a history of occupational glyphosate exposure.

## 2 Case description

The patient is a male diagnosed with chronic lymphocytic leukemia (CLL) in his mid-sixties. Two years prior to diagnosis, his first abnormal bloodwork occurred, when an elevated absolute lymphocyte count (ALC) of 4.9 × 10³/µL was noted. Eight months later, repeat bloodwork showed a similar ALC but was flagged due to a low platelet count of 146 × 10³/µL. A follow-up test revealed normalization of platelet count, and no further concerns were raised at that time.

Over the course of the following year, his white blood cell (WBC) count rose progressively, reaching 17.2 × 10³/µL. The patient had recently recovered from a severe respiratory illness and reported persistent shortness of breath for over 20 days. The leukocytosis was initially attributed to infection, although chest radiography showed no acute infiltrate or effusion.

A full two years after his initial abnormal bloodwork, WBC counts remained elevated, prompting referral to a hematologist/oncologist. Diagnostic evaluation included a manual differential, flow cytometry, and a repeat CBC with differential. The manual differential demonstrated smudge cells and atypical lymphocytes. Flow cytometry identified a monoclonal B-cell population with an absolute lymphocyte count of 8.3 × 10³/µL and an immunophenotype of CD5(+), CD19(+), CD20 (low-density), CD23/CD200/CD45(+), lambda-restricted, and CD38(–). The presence of a clonal B-cell population exceeding 5 × 10³/µL with a characteristic CLL immunophenotype supported the diagnosis of CLL rather than monoclonal B-cell lymphocytosis.

Genetic and molecular testing were subsequently performed for prognostic evaluation. TP53 sequencing revealed no mutation. FISH analysis demonstrated a 13q14 deletion (**Figure 1**) and was negative for trisomy 12, 6q deletion (**Figure 2**), ATM deletion, and TP53 deletion (17p13.1). IGHV was mutated at 12.93%. The presence of del(13q), mutated IGHV, and absence of TP53 mutation or del(17p) were consistent with favorable-risk disease and supported an anticipated indolent clinical course. The patient’s clinical course and diagnostic evaluation are summarized in **Figure 3**.

**Figure 1:**
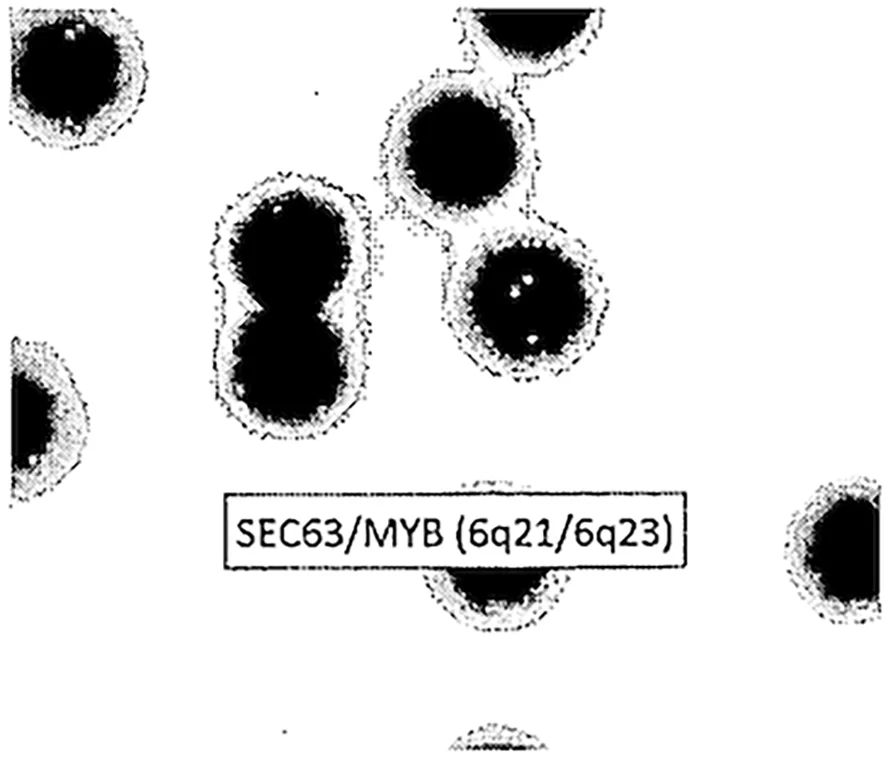
Image results from FISH B-cell chronic lymphocytic leukemia panel demonstrating positive FISH results for 13q14 deletion and negative results for trisomy 12 and deletions of 6q, ATM, and TP53 (17p13.1). Used with patient permission.

**Figure 2:**
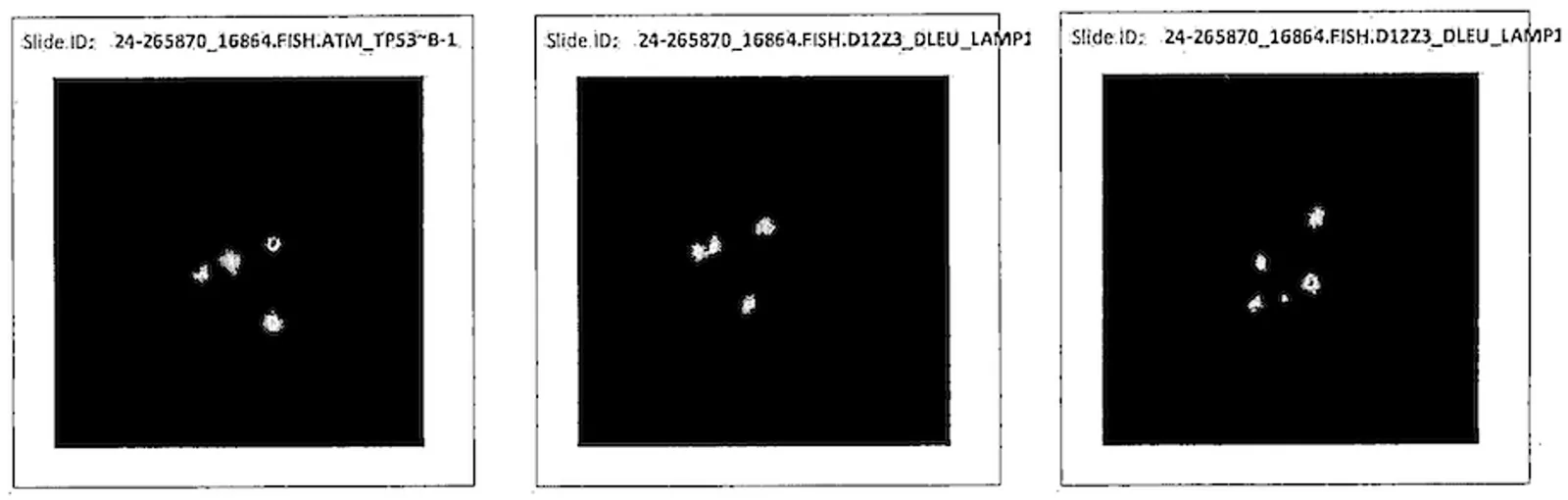
Fluorescence *in situ* hybridization (FISH) analysis of the SEC63/MYB (6q21/6q23) locus. Used with patient permission.

**Figure 3:**
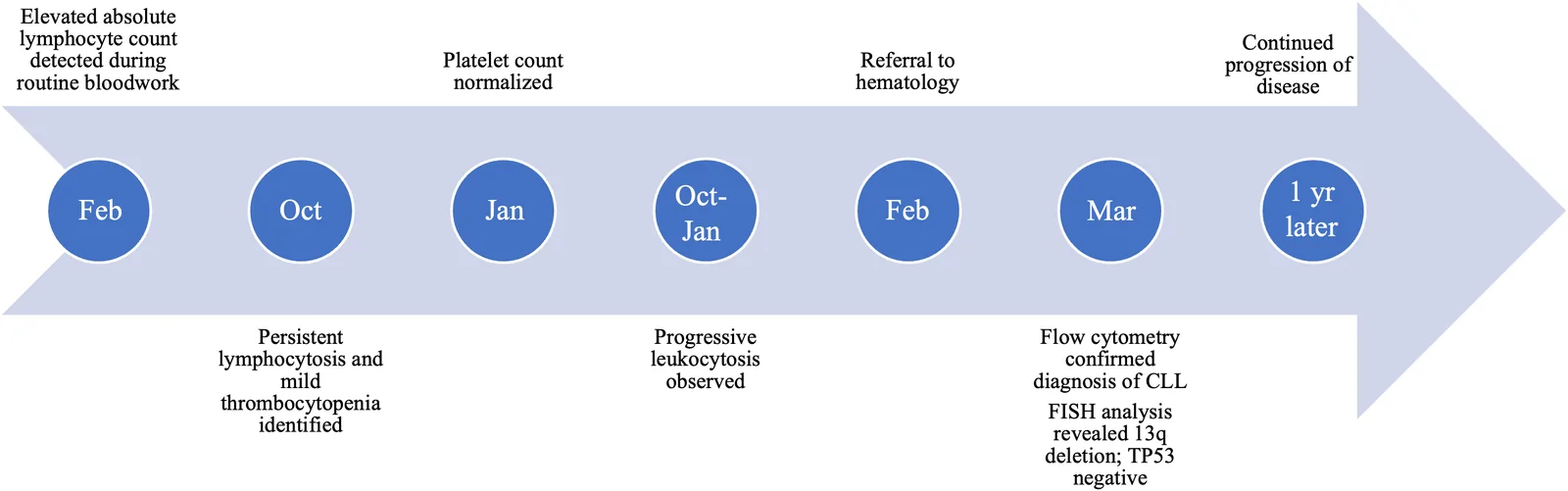
Timeline of clinical course. Overview of the patient’s symptom progression, diagnostic workup, and management.

Further history identified a family history of CLL and a separate history of occupational herbicide exposure. The patient reported 8 years of occupational exposure to glyphosate-based herbicides (Roundup^®^), with application approximately every other week during the roughly 10-month growing season in Northern California. Assuming the frequency of use, this corresponds to approximately 22 application days annually and an estimated 176 cumulative lifetime exposure days over the eight-year period. The product was diluted according to the manufacturer’s recommendation at 6 fluid ounces of product per 1 gallon of water and applied using a residential 2-gallon pump sprayer. Personal protective equipment was limited to latex gloves, with potential exposure occurring through dermal contact and inhalation. An absorbed cumulative glyphosate dose could not be estimated from the available history. The patient reported no additional occupational pesticide exposure.

His father, a Vietnam War veteran with a history of Agent Orange exposure, was also diagnosed with CLL. The patient’s father served as an Air Force loadmaster and reported occupational exposure to Agent Orange while loading the herbicide onto aircraft. Additional details regarding the frequency, duration, or cumulative extent of exposure were unavailable. The patient’s family history and occupational exposure are reported as distinct factors; their individual or combined contributions to his disease cannot be determined from this case.

### 2.1 Follow up and outcomes

Following diagnosis, the patient was managed with active surveillance and serial clinical and laboratory assessment. Serial hematologic findings from initial lymphocytosis through follow-up are summarized in **Table 1**. Over the course of follow-up, his WBC increased from 17.2 × 10³/µL at diagnosis to 42.4 × 10³/µL at the most recent evaluation (March 2025), while hemoglobin and platelet counts remained stable. He subsequently developed axillary lymphadenopathy, resulting in progression from Rai stage 0 to Rai stage I disease. No hepatosplenomegaly or progressive cytopenias were identified.

**Table 1:** Serial hematologic findings and clinical course from initial lymphocytosis through follow-up.

Date	WBC (×10³/µL)	ALC (×10³/µL)	Hgb (g/dL)	Platelets (×10³/µL)	Clinical findings
Feb 2022	7.7	4.9	15.4	175	Initial lymphocytosis
Oct 2022	8.1	4.6	15.5	146	Persistent lymphocytosis; mild thrombocytopenia
Jan 2024	14.1	10	14.1	138	Recent respiratory illness
Mar 2024	17.2	12.04	14.7	139	CLL diagnosed; Rai 0
Dec 2024	29.8	27.12	14.6	128	Progressive lymphocytosis; Rai 0
Mar 2025	42.4	37.31	14.9	147	Axillary lymphadenopathy; Rai 1

At the most recent hematology evaluation, the patient remained overall asymptomatic, with a reported weight loss of twenty pounds in four months. Despite the progressive leukocytosis and development of lymphadenopathy, he did not meet established indications for CLL-directed therapy, and active surveillance was continued. The possibility of future treatment was discussed should his leukocytosis continue to progress and symptoms develop.

## 3 Discussion

### 3.1 Genetic background

Genetics plays a significant role in disease etiology, with genetic factors estimated to account for 35% of cases. Ten percent of CLL patients have a first-degree relative who is also affected, and risk is stratified from 5–8× with one relative up to 27× with two affected first-degree relatives. Genome-wide association studies have identified 41 independent single-nucleotide polymorphisms (SNPs) that show strong statistical associations with disease, accounting for an estimated 25% of heritability. The ATM tumor suppressor gene is implicated in CLL disease progression, with approximately 20% of patients found to have an 11q deletion. Germline variants of ATM have been associated with younger age (<55) at diagnosis (5).

Cytogenetic abnormalities detected by fluorescence *in situ* hybridization (FISH) are present in more than 80% of patients with CLL: del(13q) (55%), del(11q) (18%), trisomy 12 (16%), del(17p) (7%), and del(6q) (7%). Del(13q) alone is associated with a favorable prognosis and the longest median survival (133 months) after chemotherapy. Del(11q) is associated with shorter median survival (79 months) and extensive lymphadenopathy. Del(17p) reflects TP53 mutation and predicts poor outcomes. Other mutations in ATM, NOTCH1, SF3B1, and BIRC3 predict shorter time to first treatment or overall survival (3).

This patient tested positive for a 13q deletion, which is associated with a favorable prognosis. Additionally, our patient had an immunoglobulin heavy chain variable region (IGHV) mutation rate of nearly 13%, which has been associated with improved progression-free survival (PFS) and better response to the FCR regimen.

### 3.2 Epigenetics

CLL patients can be classified into two main groups based on somatic hypermutation in the IGHV locus (6). Germline IGHV sequence is used in clinical practice to differentiate CLL subtypes. Higher deviation from the germline sequence is associated with favorable PFS and overall survival in patients treated with fludarabine, cyclophosphamide, and rituximab (FCR). IGHV-unmutated status (<2% mutation) and 17p deletion are associated with significantly shorter PFS (3).

### 3.3 Agent orange

In a large case-control study from the Veterans Affairs Million Veteran Program, genetic predisposition and Agent Orange exposure were independently associated with CLL and other lymphoid malignancies. Investigators analyzed more than 255, 000 U.S. veterans and found that Agent Orange exposure was associated with increased odds of CLL (odds ratio [OR] 1.61, 95% CI 1.40–1.84) (7). Higher polygenic risk scores were independently associated with greater odds of these malignancies, with the strongest association observed for CLL (OR 1.81, 95% CI 1.70–1.93) (7). These findings support consideration of inherited susceptibility and environmental exposure as separate risk domains. In the present case, the patient’s father had both CLL and a history of Agent Orange exposure. The father’s exposure does not establish inherited genetic susceptibility in the patient; the patient’s family history of CLL and the father’s environmental exposure should therefore be interpreted separately.

### 3.4 Glyphosate herbicides

The relationship between glyphosate-based herbicides (Roundup^®^) and CLL remains controversial. Epidemiologic studies suggest that farmers and agricultural workers with higher pesticide exposure have an increased risk for non-Hodgkin lymphomas, including CLL (5). The International Agency for Research on Cancer (IARC) classified glyphosate as a carcinogen in 2015, although the U.S. Environmental Protection Agency (EPA) did not reach the same conclusion (5). Some studies showed no statistically significant association, while others reported increased risk (OR > 2.0). A meta-analysis suggested a 41% increased risk of non-Hodgkin lymphoma overall, and up to 84% when excluding shorter-term cohorts (5). Preclinical studies provided biologic plausibility, as glyphosates are genotoxic and carcinogenic in some models, though results remained inconsistent. Collectively, the available evidence regarding glyphosate exposure and CLL remains inconclusive. The patient’s exposure raises the possibility of an environmental association, but this case cannot establish that glyphosate contributed to his disease or interacted additively with familial susceptibility.

### 3.5 Disease progression and prognosis

CLL exhibits a heterogeneous clinical course. Prognosis is guided by Rai and Binet staging systems and refined by molecular markers such as TP53, IGHV, and β_2_-microglobulin levels (8). The CLL-International Prognostic Index (CLL-IPI) integrates these to define risk groups with distinct outcomes. For early-stage asymptomatic patients, a “watch and wait” strategy remains standard, as early treatment confers no survival benefit (8). Prognosis can be refined using models such as the IPS-E for early-stage CLL, which estimates 5-year treatment risk based on IGHV status, lymphocyte count, and nodal involvement (8). Targeted agents such as BTK and BCL2 inhibitors have improved outcomes, though high-risk molecular features still predict more aggressive disease (8). Although causality or interaction between risk factors cannot be established from a single case, this report highlights the need for continued investigation of familial risk and environmental exposures as factors potentially associated with hematologic malignancies.

### 3.6 Limitations

As a retrospective assessment of a single patient, the available clinical history was limited in part by recall bias, as certain aspects of patient presentation, symptoms and disease course relies on self-reported information from the patient. Additionally, the single-patient design limits the generalizability of these findings and prevents definitive conclusions of causality.

## 4 Conclusion

This case describes CLL diagnosed earlier than the reported median age in a patient with a family history of CLL and a concomitant history of occupational glyphosate exposure. Current evidence regarding glyphosate and CLL remains inconclusive, and this case cannot determine whether the exposure contributed to disease development or age at diagnosis. Similarly, the father’s Agent Orange exposure should not be interpreted as evidence of inherited genetic susceptibility in the patient. The patient’s family history and occupational exposure are therefore best understood as distinct epidemiologic observations that raise questions for future study. His molecular findings, including del(13q) and mutated IGHV status, provide clinically established prognostic information, although individual disease courses remain variable. Further research is needed to clarify the genetic and environmental factors associated with CLL.

## 5 Patient perspective

“The realization that I have Chronic Lymphocytic Leukemia (CLL) did not penetrate my mental acceptance that day while sitting in the doctor’s office with my wife, listening to the oncologist explain the complex workings of a blood disease. Nor did it sink in for several days, if not weeks. Not until I immersed myself in the pathology and cause and effect did I come to realize that my Complete Blood Count (CBC) “bad” numbers were not an anomaly or an acute infection that would render itself harmless, but rather a sign of a disease that would slowly kill me.

However, interestingly enough, should another calamity not befall me, and based on my current CBC numbers, I should live to my statistical age. Not a horrible cancer diagnosis to have on the cancer spectrum, but I imagine within that time, the disease will cause disruptions and discomfort in my life requiring increased intervention. Currently, based on my CBC, I am on a watch and wait treatment plan; meaning, absent any physical signs attributed to the disease such as swollen lymph nodes, extreme fatigue, enlarged spleen, bruising and night sweats to name a few, there is no treatment. The possibility that the disease has advanced is only my next blood test away.

I will never feel accustomed to having CLL. My blood numbers will never decline, and I know over time I will start presenting outward symptoms necessitating the need for increased care. But for now, all I can do is live my life.”

## Data Availability

The original contributions presented in the study are included in the article/supplementary material. Further inquiries can be directed to the corresponding author.
